# In Silico Identification of Key Genes and Immune Infiltration Characteristics in Epicardial Adipose Tissue from Patients with Coronary Artery Disease

**DOI:** 10.1155/2022/5610317

**Published:** 2022-10-29

**Authors:** Yisen Deng, Xuming Wang, Zhan Liu, Xiaoshuo Lv, Bo Ma, Qiangqiang Nie, Xueqiang Fan, Yuguang Yang, Zhidong Ye, Peng Liu, Jianyan Wen

**Affiliations:** ^1^Peking University China-Japan Friendship School of Clinical Medicine, Beijing, China; ^2^Department of Cardiovascular Surgery, China-Japan Friendship Hospital, Beijing, China

## Abstract

**Background:**

The present study is aimed at identifying the differentially expressed genes (DEGs) and relevant biological processes and pathways associated with epicardial adipose tissue (EAT) from patients with coronary artery disease (CAD). We also explored potential biomarkers using two machine-learning algorithms and calculated the immune cell infiltration in EAT.

**Materials and Methods:**

Three datasets (GSE120774, GSE64554, and GSE24425) were obtained from the Gene Expression Omnibus (GEO) database. The GSE120774 dataset was used to evaluate DEGs between EAT of CAD patients and the control group. Functional enrichment analyses were conducted to study associated biological functions and mechanisms using the Kyoto Encyclopedia of Genes and Genomes (KEGG), Gene Ontology (GO), and Gene Set Enrichment Analysis (GSEA). After this, the least absolute shrinkage and selection operator (LASSO) and support vector machine recursive feature elimination (SVM-RFE) were performed to identify the feature genes related to CAD. The expression level of the feature genes was validated in GSE64554 and GSE24425. Finally, we calculated the immune cell infiltration and evaluated the correlation between the feature genes and immune cells using CIBERSORT.

**Results:**

We identified a total of 130 upregulated and 107 downregulated genes in GSE120774. Functional enrichment analysis revealed that DEGs are associated with several pathways, including the calcium signaling pathway, complement and coagulation cascades, ferroptosis, fluid shear stress and atherosclerosis, lipid and atherosclerosis, and regulation of lipolysis in adipocytes. TCF21, CDH19, XG, and NNAT were identified as feature genes and validated in the GSE64554 and GSE24425 datasets. Immune cell infiltration analysis showed plasma cells are significantly more numerous in EAT than in the control group (*p* = 0.001), whereas macrophage M0 (*p* = 0.024) and resting mast cells (*p* = 0.036) were significantly less numerous. TCF21, CDH19, XG, and NNAT were correlated with immune cells, including plasma cells, M0 macrophages, and resting mast cells.

**Conclusion:**

TCF21, CDH19, XG, and NNAT might serve as feature genes for CAD, providing new insights for future research on the pathogenesis of cardiovascular diseases.

## 1. Introduction

Coronary artery disease (CAD) is one of the leading causes of death worldwide, and atherosclerosis is its most basic associated pathophysiological change [1]. Obesity represents a significant risk factor for cardiovascular disease, and the expansion of ectopic and visceral fat is strongly involved in the pathogenesis of CAD [2]. Recent evidence revealed the promising role of epicardial adipose tissue (EAT) in the occurrence, development, and prognosis of CAD [3]. EAT is recognized as a unique adipose storage, supplied by the branches of the coronary artery and directly adjacent to the myocardium. It is mainly comprised of adipocytes, stroma-vascular cells, fibroblasts, nerves, and various immune cells. Besides providing energy storage, the EAT serves as an endocrine and immune organ [4, 5]. Under physiological conditions, the EAT plays an important part in cardiac metabolism, prevention of cardiac lipotoxicity, mechanical protection of coronary arteries, and provides immunological support for the heart [6]. The link between EAT inflammation and CAD has increasingly attracted research focus. Over the recent years, the EAT has been proposed as a biomarker for acute coronary syndrome (ACS), major adverse cardiac events (MACE), and atrial fibrillation (AF) [7–9]. Moreover, several large-scale cohort studies demonstrated that the EAT volume is positively associated with the occurrence, development, and prognosis of CAD [10–12]. Specifically, it is currently accepted that some cytokines secreted by the EAT either protect or negatively affect cardiomyocytes' function and coronary arteries through paracrine or vasocrine mechanisms [13, 14]. Cytokines secreted by the EAT might diffuse through the interstitial fluid into coronary wall layers. Besides, they could be directly released into the vasa vasorum of the coronary arteries [15, 16]. In pathological conditions, the proinflammatory or proatherogenic factors secreted by the EAT, including IL-6, IL-8, monocyte chemoattractant protein 1, leptin, resistin, and tumor necrosis factor *α* [15], exert their pathophysiological effects through direct diffusion, enhancing the potential to induce atherogenic changes in monocytes and endothelial cells [17]. Leptin, for example, is regarded as an independent risk factor for atherosclerosis that exerts a variety of atherogenic effects, such as increasing endothelial dysfunction, promoting inflammatory responses, oxidative stress induction, platelet aggregation and migration, and the proliferation of vascular smooth muscle cells [3, 18].

Although a high number of studies confirmed the involvement of the EAT in the development and progression of coronary atherosclerosis through adipokines, the exact mechanisms through which the EAT participates in CAD remain unclear [3, 5, 19–21]. A considerable limitation of these studies relates to the sole recruitment of patients who underwent cardiac surgery. Furthermore, it is difficult to collect the EAT from healthy subjects due to ethical concerns, whereby the subcutaneous adipose tissue (SAT) is usually used as control across various studies [22–25]. Bioinformatics analysis has been extensively applied to the identification of differentially expressed genes (DEGs) at the genome-wide level and constitutes a useful strategy for exploring the potential biomarkers and molecular mechanisms associated with the EAT and CAD. Here, we screened two microarray datasets from the Gene Expression Omnibus (GEO) database for DEGs between the EAT and the SAT. We attempted to explore the underlying biological functions using enrichment analysis and identified the best feature genes by employing machine-learning algorithms. In addition, we used CIBERSORT to investigate the proportion of immune cells that are present in the EAT [26, 27] and studied the relationship between the feature genes and infiltrating immune cells to provide a basis for further research.

## 2. Materials and Methods

### 2.1. Microarray Data

The GSE120774, GSE64554, and GSE24425 datasets were downloaded from the GEO database (https://www.ncbi.nlm.nih.gov/geo/). The GSE120774 dataset was used as the discovery cohort, and GSE64554 and GSE24425 datasets were used as the validation cohort. We analyzed a total of 9 EAT and 8 SAT samples from patients with CAD in GSE120774, which was based on the GPL6244 Affymetrix Human Gene 1.0 ST Array. In addition, there were 13 EAT and 13 SAT samples from patients with CAD in GSE64554, which was based on the GPL6947 Illumina HumanHT-12 V3.0 expression bead chip. Furthermore, 6 EAT and 6 SAT samples from patients with CAD in GSE24425 were also analyzed, which was based on the GPL6884 Illumina HumanWG-6 V3.0 expression beadchip. We used the *limma* package in R to normalize the expression data and ensure a similar distribution among these datasets.

### 2.2. Identification of Differentially Expressed Genes

The DEGs were identified by the *limma* package in R. A volcano plot was used to assess the DEGs, and the cutoff was set as |log2 fold change (FC)| ≥ 1 (adjusted *p* value < 0.05).

### 2.3. Functional Annotation for Differentially Expressed Genes

Kyoto Encyclopedia of Genes and Genomes (KEGG) and Gene Ontology (GO) enrichment analyses were conducted using the Database for Annotation, Visualization, and Integrated Discovery (DAVID). GO was composed of biological processes (BP), cell components (CC), and molecular function (MF). The R package *ggplot* was used to visualize the results. Functional enrichment analysis on all expression data was performed by Gene Set Enrichment Analysis (GSEA). The R packages *clusterProfiler* and http://org.Hs.eg.db were used to conduct GSEA. The GSEA cutoff point was set as a *p* value < 0.05 and |normalized enrichment score (NES)| > 1.

### 2.4. Feature Genes Identification

We used two machine-learning algorithms to screen for the most significant candidate biomarkers between SAT and EAT. The least absolute shrinkage and selection operator (LASSO), which was based on a regression analysis algorithm, is suitable for both linear and nonlinear cases. We used the *glmnet* package in R to perform LASSO. Support vector machine (SVM) is another machine-learning algorithm that is used for regression or classification. To avoid overfitting, the SVM-recursive feature elimination (RFE) was used to screen for feature genes from selected genes. We selected the top 20 genes for the SVM-RFE algorithm according to |log2 fold change (FC)| and then merged the obtained genes using the two algorithms to get the intersection. Both LASSO and SVM-RFE were performed using the *e1071* and *mlbench* R packages. To further evaluate the diagnostic ability of the candidate biomarkers, we calculated the area under the curve (AUC) of the receiver operating characteristic (ROC) curve.

### 2.5. Immune Cell Infiltration Analysis

We used CIBERSORT (https://cibersortx.stanford.edu/) to analyze immune cell infiltration in GSE120774 and obtained 22 types of immune cells. The cutoff point was set as a *p* value < 0.05. The *vioplot* package in R was used to visualize the different immune cells in the SAT and the EAT. We also built a bar plot in R to show the percentage of immune cells present in each sample.

### 2.6. Correlation Analysis between Biomarkers and Infiltrating Immune Cells

The relationship between feature genes and immune cells was evaluated using Spearman's rank correlation analysis in R. The *ggplot*2 package was used to visualize the results.

### 2.7. Statistical Analysis

R software (version 4.2.0) was used for all statistical analyses. Continuous variables are expressed as the mean ± SD, and group comparisons were performed using Student's *t*-test for normally distributed variables and the Mann–Whitney *U* test for abnormally distributed variables. A *p* value < 0.05 was considered statistically significant.

## 3. Results

### 3.1. Identification of DEGs

**Figure 1:**
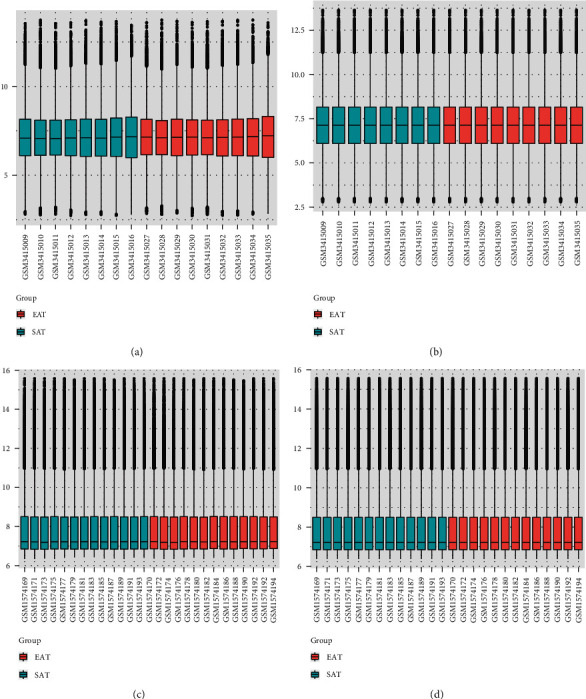
Box plot of datasets before and after normalization. GSE120774 expression profile before (a) and after (b) normalization; GSE64554 expression profile before (c) and after (d) normalization.

**Figure 2:**
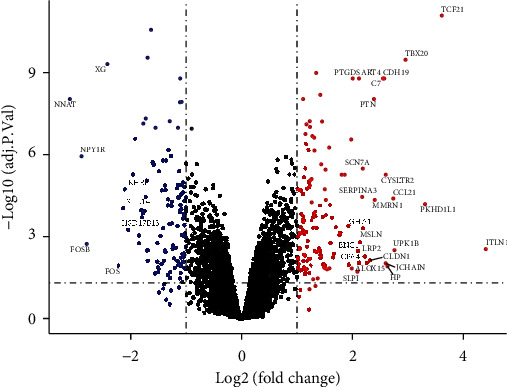
Volcano map showing the DEGs between EAT and SAT in CAD patients. The red dots represent for the upregulated genes, and the blue dots represent for downregulated genes. Genes with the most significant logFC are labeled.

The GSE120774, GSE64554, and GSE24425 datasets were normalized before analysis (Figure 1 and Supplemental File-Figure 1 show both the nonnormalized and normalized data). We identified a total of 130 upregulated and 107 downregulated genes. Genes with the most significant logFC in EAT compared with SAT in CAD patients are shown in the volcano plot of Figure 2.

### 3.2. Functional Enrichment Analysis of DEGs

**Figure 3:**
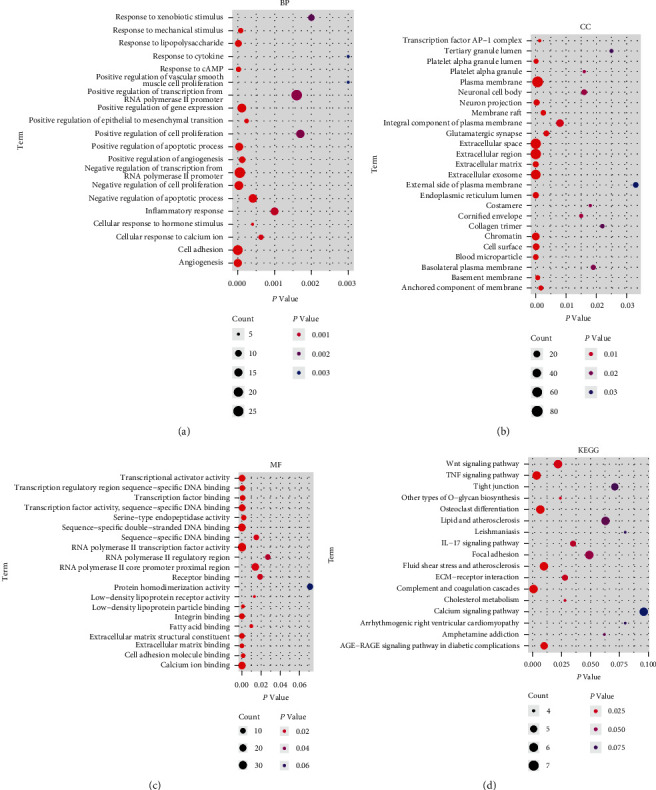
KEGG and GO functional enrichment analyses. (a) BP enrichment; (b) CC enrichment; (c) MF enrichment; (d) KEGG enrichment.

**Figure 4:**
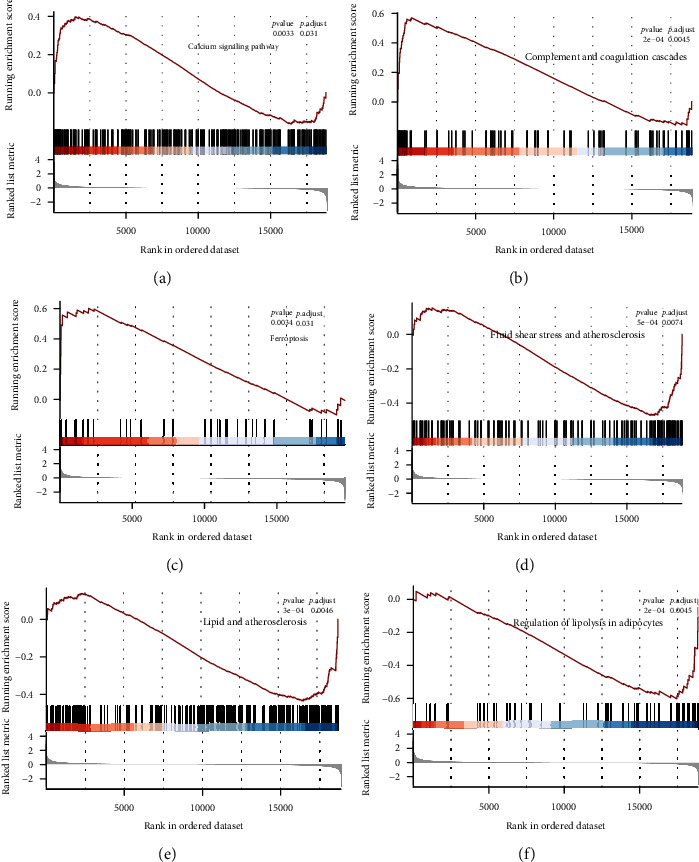
GSEA functional enrichment analysis. (a) Calcium signaling pathway; (b) complement and coagulation cascades; (c) ferroptosis; (d) fluid shear stress and atherosclerosis; (e) lipid and atherosclerosis; (f) regulation of lipolysis in adipocytes.

We subsequently conducted functional enrichment analyses, including GO, KEGG, and GSEA, to explore the biological function and pathways associated with the DEGs. GO enrichment analysis revealed that negative regulation of transcription from RNA polymerase II promoter, negative regulation of cell proliferation, cell adhesion, angiogenesis, and response to lipopolysaccharide are enriched terms in BP (Figure 3(a)); plasma membrane, extracellular space, extracellular region, and extracellular exosome are enriched terms in CC (Figure 3(b)); and RNA polymerase II transcription factor activity, calcium ion binding, integrin binding, and DNA-binding activities are enriched in MF (Figure 3(c)). In addition, KEGG pathway analysis revealed that DEGs are mainly involved in the complement and coagulation cascades, fluid shear stress and atherosclerosis, and TNF signaling pathway (Figure 3(d)). In the GSEA, we identified several enriched pathways, including the calcium signaling pathway, complement and coagulation cascades, ferroptosis, fluid shear stress and atherosclerosis, lipid and atherosclerosis, and regulation of lipolysis in adipocytes (Figures 4(a)–4(f)).

### 3.3. Identification and Validation of Feature Genes

**Figure 5:**
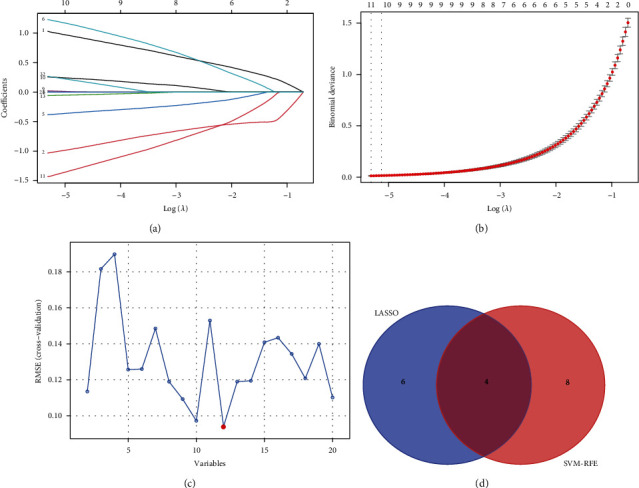
Identification of feature genes by LASSO regression and the SVM-RFE algorithm. (a) Coefficient profiles of the feature genes in the LASSO model. (b) Ten nonzero coefficients were obtained using optimal lambda (*λ*). (c) Selection of feature genes via the SVM-RFE algorithm. (d) Venn diagram showing the overlap of feature genes from the two algorithms.

**Figure 6:**
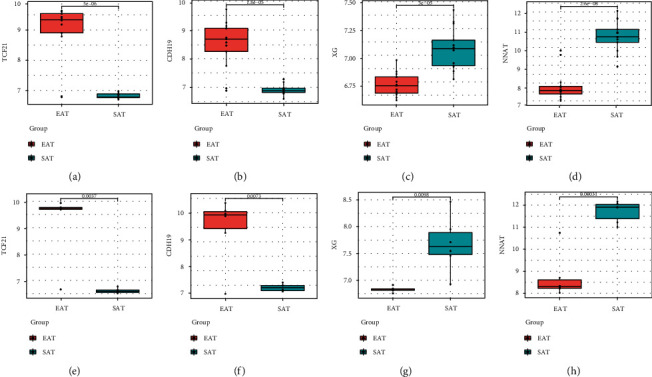
The validation of expression levels of the four feature genes in the GSE64554 (a–d) and GSE24425 (e–h) dataset. TCF21 and CDH19 were upregulated in EAT compared with SAT in CAD patients, while XG and NNAT were downregulated. (a, d) TCF21; (b, e) CDH19; (c, g) XG; (d, h) NNAT.

**Figure 7:**
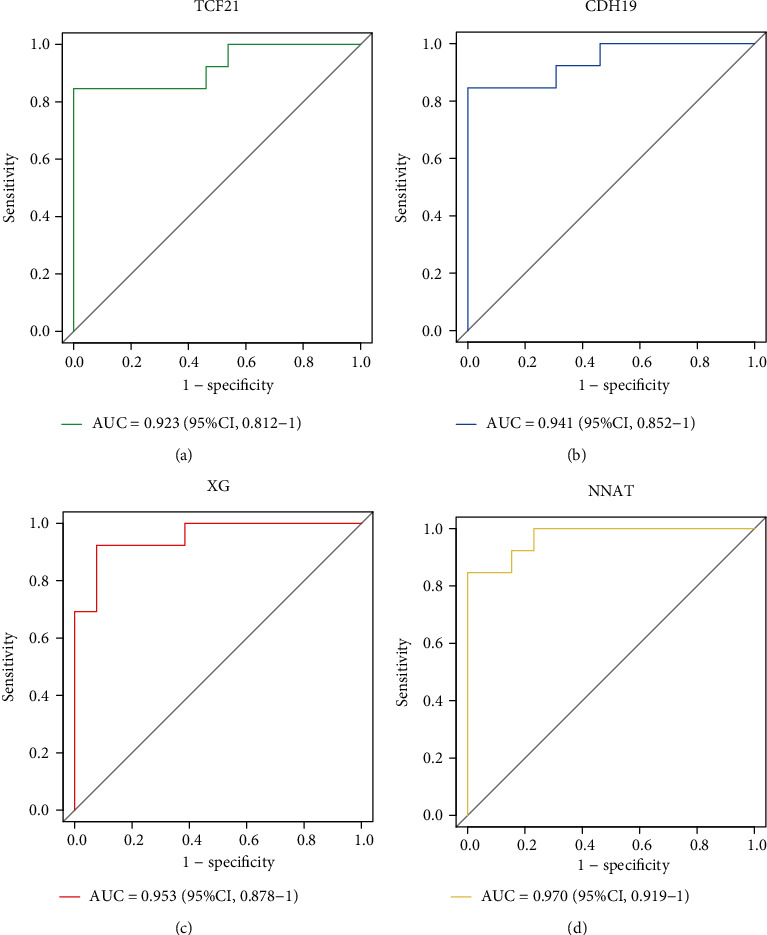
The receiver operating characteristic (ROC) curve of the four feature genes: (a) TCF21; (b) CDH19; (c) XG; (d) NNAT.

The LASSO regression algorithm was used to narrow down the number of DEGs, and 10 genes were then identified (Figures 5(a) and 5(b)). Moreover, 12 genes were obtained using the SVM-RFE algorithm (Figure 5(c)), of which 4 were also identified by LASSO (Figure 5(d)): TCF21, CDH19, XG, and NNAT. The GSE64554 and GSE24425 dataset confirmed that TCF21 and CDH19 were upregulated in EAT compared with SAT in CAD patients, whereas XG and NNAT were downregulated (Figures 6(a)–6(h)). After this, we performed ROC analysis to evaluate the diagnostic ability of these four genes in the GSE64554 dataset and found that the four feature genes have high diagnostic effectiveness in discriminating EAT from the SAT samples, with an AUC of 0.923 (95% CI = 0.812 − 1) in TCF21, 0.941 (95% CI = 0.852 − 1) in CDH19, 0.953 (95% CI = 0.878 − 1) in XG, and 0.970 (95% CI = 0.919 − 1) in NNAT (Figures 7(a)–7(d)).

### 3.4. Immune Cell Infiltration

**Figure 8:**
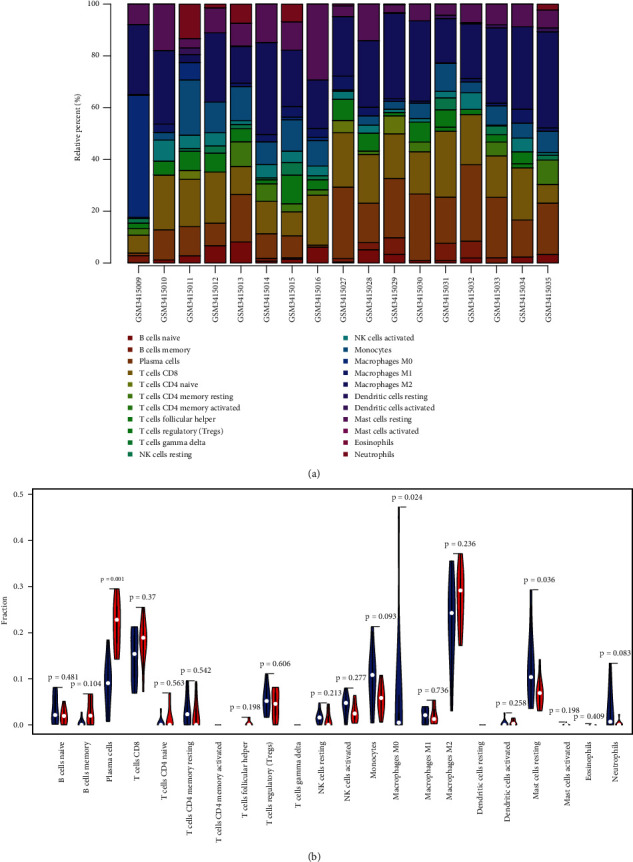
The immune cell infiltration between EAT and SAT in CAD patients. (a) The composition of 22 types of immune cells. (b) The 22 immune cell subtypes were compared between the EAT and the SAT group. The blue and red colors represent the SAT and the EAT samples, respectively.

Functional enrichment analysis revealed that DEGs might be involved in immune response, whereby we used the CIBERSORT algorithm to explore immune cell infiltration between EAT and SAT in CAD patients. The composition of immune cells in EAT vs. SAT samples in CAD patients is shown in Figure 8(a), which shows the proportions of plasma cells are notably higher in the EAT compared to the SAT (*p* = 0.001). In contrast, the proportion of M0 macrophages (*p* = 0.024) and mast cell resting (*p* = 0.036) are notably lower in the EAT than in the SAT (Figure 8(b)).

### 3.5. Correlation Analysis between the Four Feature Genes and Immune Cells

**Figure 9:**
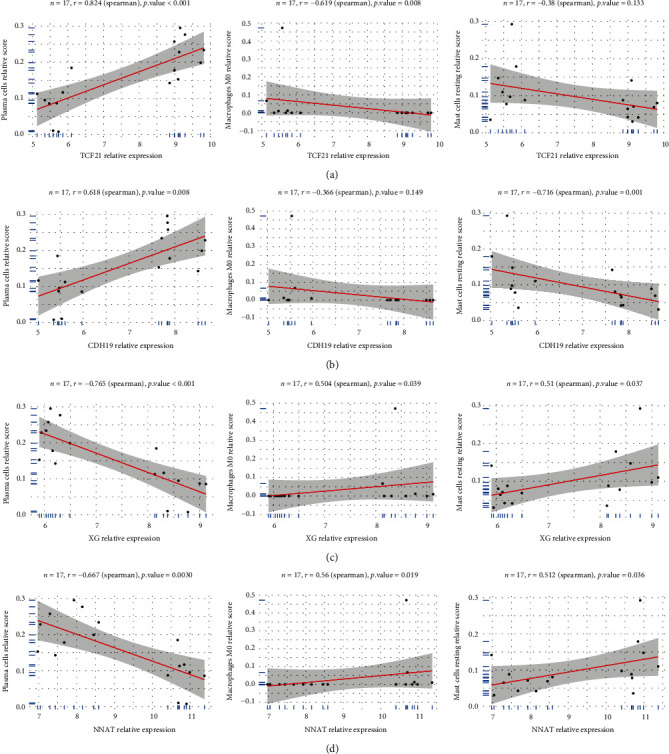
Correlation between TCF21, CDH19, XG, NNAT, and immune cells. (a) TCF21; (b) CDH19; (c) XG; (d) NNAT.

We found that TCF21 is positively correlated with plasma cells (*r* = 0.824, *p* < 0.001), but negatively correlated with M0 macrophages (*r* = −0.619, *p* = 0.008), while CDH19 is positively correlated with plasma cells (*r* = 0.618, *p* = 0.008), and negatively correlated with resting mast cells (*r* = −0.716, *p* = 0.001). In addition, XG is positively correlated with M0 macrophages (*r* = 0.504, *p* = 0.039) and resting mast cells (*r* = 0.51, *p* = 0.037), and negatively correlated with plasma cells (*r* = −0.765, *p* < 0.001). Finally, NNAT is positively correlated with M0 macrophages (*r* = 0.56, *p* = 0.019) and resting mast cells (*r* = 0.512, *p* = 0.036) and negatively correlated with plasma cells (*r* = −0.667, *p* = 0.003) (Figures 9(a)–9(d)). Overall, we found that the four feature genes are highly correlated with immune cells.

## 4. Discussion

The EAT participates in the pathological process of atherosclerosis through the endocrine and paracrine pathways, although the specific mechanisms remain unknown [14]. Here, we found 130 upregulated and 107 downregulated genes from a microarray analysis. Functional enrichment analysis indicated that these DEGs are involved in various pathophysiological processes and that four feature genes (TCF21, CDH19, XG, and NNAT) identified via LASSO regression and the SVM-RFE algorithm are correlated with immune cells, including plasma cells, M0 macrophages, and resting mast cells, as shown by infiltration analysis.

Previous studies have revealed that adipokines secreted by the EAT might affect myocardial cells and coronary arteries [3, 19]. Hypoxic and dysfunction of EAT might lead to lipolysis and inflammatory activities through the dysregulated secretion of vasoactive and inflammatory factors, which are involved in the process of atherosclerosis, including vascular remodeling, endothelial dysfunction, the proliferation and migration of smooth muscle cell (SMC), foam cell formation, and plaque destabilization [28]. Intelectin 1 (ITLN1), which in our analysis had the highest expression differences between the EAT and the SAT (Figure 2, Supplemental File-Figure 2A), is abundantly expressed in visceral adipose tissue and known to regulate obesity-related cardiometabolic disorders through its anti-inflammatory activity [29]. Leptin is regarded as an independent risk factor for atherosclerosis that exerts a variety of atherogenic effects. However, the expression level of leptin was not significantly higher in EAT compared with SAT in our analysis (Supplemental File–Figure 2(b)), and we hypothesize that the reasons might be as follows: (1) the samples are not sufficient to show significant differences; (2) leptin in EAT might mainly derived from circulation. In contrast, chemerin, which can bind to the G protein-coupled receptor (CMKLR1), is associated with immune response and the metabolism of glucose and lipids [30], and its expression levels are reportedly positively associated with coronary atherosclerosis [21].

Our study identified four feature genes (TCF21, CDH19, XG, and NNAT) associated with CAD using two machine-learning algorithms. TCF21 is involved in cardiac fibrosis and plays a critical role in the fate of smooth muscle cells [31], promoting SMC dedifferentiation by inhibiting the serum response factor-myocardin axis (SRF-MYOCD) [32]. The specific effects of TCF21 on atherosclerosis are complex. On the one hand, TCF21 suppresses the progression of atherosclerosis by regulating the transition from SMC to fibromyocytes and promoting the formation of antiatherosclerotic fibrous caps on the lesions [33]. On the other hand, when compared with the control, the transfection of TCF21 siRNA (siTGF21) notably decreases the level of reactive oxygen species (ROS) and cell apoptosis-related protein Bax and leads to an increase in the expression of active antiapoptotic protein Bcl-2 in human umbilical vein cells (HUVECs) [34]. This suggests that TCF21 might promote atherosclerosis via increasing the apoptosis rate and ROS accumulation. Cadherin 19 (CDH19) is a gene encoding calcium-dependent cell adhesion proteins involved in vascular remodeling and plays a critical role in the structural integrity of blood vessels [35]. Recent studies have demonstrated the involvement of classic cadherin in many complex processes, such as angiogenesis, morphogenesis, cellular communication, and cellular proliferation [36–38]. Niu et al. [39] revealed that the expression knockdown of CDH12 and CDH19 markedly inhibits monocyte chemotactic protein-1-induced protein (MCPIP) and suppresses the capillary-like tube formation in HUVECs. Moreover, CDH19 might serve as a new target of tumorigenesis and drug development for glioblastoma stem-like cells (GSC) and can be considered an independent prognostic biomarker of lung adenocarcinoma (LUAD) and breast cancer (BC) [36, 40, 41]. XG was one of the blood group systems located at the pseudoautosomal boundary on the short arm of chromosome X, composed of two X-borne alleles, Xg a and Xg [42]. Recent studies evaluating the biological functions of the gene were limited to its association with red blood cells (RBC). Meynet et al. showed that high XG protein expression in Ewing's sarcoma (EWS) is associated with a worse prognosis. Furthermore, the overexpression of XG increased the proliferation and migration of EWS cells in vitro, while the knockdown of the gene with short hairpin RNA led to the opposite effect [43]. However, the role played by XG in atherosclerosis remains uncharacterized. Finally, NNAT is a paternally imprinted gene, which is expressed in the developing brain, pituitary, pancreas, and adipose tissue, and plays an important role in the appetite behavior, energy balance, adipogenesis, and inflammatory responses associated with insulin resistance [44–46]. Gene set enrichment analysis indicated a significantly negative correlation exists between NNAT and energy metabolism, but uncovered a positive correlation with inflammation [46]. It has been reported that NNAT inhibits oxidative stress and inflammation and promotes adipocyte differentiation by mediating the NF-*κ*B signal pathway [45]. NNAT expression levels are also closely associated with endothelial dysfunction and EAT secretion [45, 47]. Furthermore, it has been found that increased NNAT expression levels are associated with poor prognosis in myxoid liposarcoma, lung cancer, and breast cancer [48–50]. However, very few studies clarified the association of this gene with atherosclerosis.

We calculated immune cell infiltration and estimated the correlation between the four genes and immune cells. We found that the four feature genes are correlated with immune cells, including plasma cells, M0 macrophages, and resting mast cells. To our knowledge, this is the first study to calculate the infiltration of the immune cells in EAT vs. SAT. Adipocytes not only serve as an energy storage depot but also play a critical role in endocrine and immune. Adipokines, such as leptin and adiponectin, are critical for the development of B cells, activation, and antibody production [51]. Hence, adipocytes play a crucial role in adaptive immunity mediated by B cells.

Despite the associations described above, few studies investigating the molecular mechanisms between these four genes and immune cells have been published to date, whereby further experiments are required to explore their pathogenesis. Among the limitations to our study, we can include (1) the choice of the SAT as control rather than the EAT of healthy individuals (due to ethical restrictions). Hence, the difference between the EAT and the SAT in healthy groups remains unknown; (2) the three datasets have limited sample sizes; (3) the association between the feature genes and CAD and their interaction with immune cells needs further investigation on larger sample sizes to confirm our observations.

## 5. Conclusions

In this study, we identified the DEGs between the EAT and the SAT in patients with CAD and explored the potential biological processes and pathways involved. The identified DEGs are mainly associated with the calcium signaling pathway, complement and coagulation cascades, ferroptosis, fluid shear stress and atherosclerosis, lipid and atherosclerosis, and regulation of lipolysis in adipocytes. In addition, the four feature genes identified (TCF21, CDH19, XG, and NNAT) might serve as feature genes for CAD, bringing new insights into the pathogenesis of cardiovascular diseases.

## Data Availability

All the analyses in this study were based on the publicly available datasets (GSE120774, GSE64554, and GSE24425). Original data are available in the GEO database (https://www.ncbi.nlm.nih.gov/).

## Supplementary Materials

Supplementary Materials Supplemental File Figure 1: box plot of datasets before and after normalization. GSE24425 expression profile before (A) and after (B) normalization. Supplemental File Figure 2: the expression levels of the ITLN1 and leptin in the GSE120774. (A) ITLN1; (B) leptin.

## References

[1] Boudoulas K. D., Triposciadis F., Geleris P., Boudoulas H. (2016). Coronary atherosclerosis: pathophysiologic basis for diagnosis and management. *Progress in Cardiovascular Diseases*.

[2] Mazzotta C., Basu S., Gower A. C. (2021). Perivascular adipose tissue inflammation in ischemic heart disease. *Arteriosclerosis, Thrombosis, and Vascular Biology*.

[3] Zhang T., Yang P., Li T., Gao J., Zhang Y. (2019). Leptin expression in human epicardial adipose tissue is associated with local coronary atherosclerosis. *Medical Science Monitor*.

[4] Huh J. Y., Park Y. J., Ham M., Kim J. B. (2014). Crosstalk between adipocytes and immune cells in adipose tissue inflammation and metabolic dysregulation in obesity. *Molecules and Cells*.

[5] Mancio J., Oikonomou E. K., Antoniades C. (2018). Perivascular adipose tissue and coronary atherosclerosis. *Heart*.

[6] Antonopoulos A. S., Antoniades C. (2017). The role of epicardial adipose tissue in cardiac biology: classic concepts and emerging roles. *The Journal of Physiology*.

[7] Mahabadi A. A., Lehmann N., Kälsch H. (2014). Association of epicardial adipose tissue and left atrial size on non-contrast CT with atrial fibrillation: the Heinz Nixdorf recall study. *European Heart Journal Cardiovascular Imaging*.

[8] Nakanishi K., Fukuda S., Tanaka A. (2014). Persistent epicardial adipose tissue accumulation is associated with coronary plaque vulnerability and future acute coronary syndrome in non-obese subjects with coronary artery disease. *Atherosclerosis*.

[9] Forouzandeh F., Chang S. M., Muhyieddeen K. (2013). Does quantifying epicardial and intrathoracic fat with noncontrast computed tomography improve risk stratification beyond calcium scoring alone?. *Circulation. Cardiovascular Imaging*.

[10] Mahabadi A. A., Massaro J. M., Rosito G. A. (2008). Association of pericardial fat, intrathoracic fat, and visceral abdominal fat with cardiovascular disease burden: the Framingham heart study. *European Heart Journal*.

[11] Mcclain J., Hsu F., Brown E. (2013). Pericardial adipose tissue and coronary artery calcification in the multi- ethnic study of atherosclerosis (MESA). *Obesity*.

[12] Mahabadi A. A., Lehmann N., Kälsch H. (2014). Association of epicardial adipose tissue with progression of coronary artery calcification is more pronounced in the early phase of atherosclerosis: results from the Heinz Nixdorf recall study. *JACC: Cardiovascular Imaging*.

[13] Cherian S., Lopaschuk G. D., Carvalho E. (2012). Cellular cross-talk between epicardial adipose tissue and myocardium in relation to the pathogenesis of cardiovascular disease. *American Journal of Physiology. Endocrinology and Metabolism*.

[14] D'Marco L., Puchades M. J., Gorriz J. L. (2020). Epicardial adipose tissue, adiponectin and leptin: a potential source of cardiovascular risk in chronic kidney disease. *International Journal of Molecular Sciences*.

[15] Madonna R., Massaro M., Scoditti E., Pescetelli I., De Caterina R. (2019). The epicardial adipose tissue and the coronary arteries: dangerous liaisons. *Cardiovascular Research*.

[16] Yudkin J. S., Eringa E., Stehouwer C. D. (2005). "Vasocrine" signalling from perivascular fat: a mechanism linking insulin resistance to vascular disease. *Lancet*.

[17] Karastergiou K., Evans I., Ogston N. (2010). Epicardial adipokines in obesity and coronary artery disease induce atherogenic changes in monocytes and endothelial cells. *Arteriosclerosis, Thrombosis, and Vascular Biology*.

[18] Katsiki N., Mikhailidis D. P., Banach M. (2018). Leptin, cardiovascular diseases and type 2 diabetes mellitus. *Acta Pharmacologica Sinica*.

[19] Du Y., Ji Q., Cai L. (2016). Association between omentin-1 expression in human epicardial adipose tissue and coronary atherosclerosis. *Cardiovascular Diabetology*.

[20] Zhou Y., Wei Y., Wang L. (2011). Decreased adiponectin and increased inflammation expression in epicardial adipose tissue in coronary artery disease. *Cardiovascular Diabetology*.

[21] Gao X., Mi S., Zhang F. (2011). Association of chemerin mRNA expression in human epicardial adipose tissue with coronary atherosclerosis. *Cardiovascular Diabetology*.

[22] Gruzdeva O. V., Akbasheva O. E., Dyleva Y. A. (2017). Adipokine and cytokine profiles of epicardial and subcutaneous adipose tissue in patients with coronary heart disease. *Bulletin of Experimental Biology and Medicine*.

[23] Vacca M., Di Eusanio M., Cariello M. (2016). Integrative miRNA and whole-genome analyses of epicardial adipose tissue in patients with coronary atherosclerosis. *Cardiovascular Research*.

[24] Hirata Y., Tabata M., Kurobe H. (2011). Coronary atherosclerosis is associated with macrophage polarization in epicardial adipose tissue. *Journal of the American College of Cardiology*.

[25] Mráz M., Cinkajzlová A., Kloučková J. (2019). Dendritic cells in subcutaneous and epicardial adipose tissue of subjects with type 2 diabetes, obesity, and coronary artery disease. *Mediators of Inflammation*.

[26] Zhao E., Xie H., Zhang Y. (2020). Predicting diagnostic gene biomarkers associated with immune infiltration in patients with acute myocardial infarction. *Frontiers in Cardiovascular Medicine*.

[27] Zeng H., Liu X., Zhang Y. (2021). Identification of potential biomarkers and immune infiltration characteristics in idiopathic pulmonary arterial hypertension using bioinformatics analysis. *Frontiers in Cardiovascular Medicine*.

[28] Miksztowicz V., Morales C., Barchuk M. (2017). Metalloproteinase 2 and 9 activity increase in epicardial adipose tissue of patients with coronary artery disease. *Current Vascular Pharmacology*.

[29] Tan Y. L., Zheng X. L., Tang C. K. (2015). The protective functions of omentin in cardiovascular diseases. *Clinica Chimica Acta*.

[30] Wittamer V., Franssen J. D., Vulcano M. (2003). Specific recruitment of antigen-presenting cells by chemerin, a novel processed ligand from human inflammatory fluids. *The Journal of Experimental Medicine*.

[31] Acharya A., Baek S. T., Huang G. (2012). The bHLH transcription factor Tcf21 is required for lineage-specific EMT of cardiac fibroblast progenitors. *Development*.

[32] Xie Y., Martin K. A. (2020). TCF21: flipping the phenotypic switch in SMC. *Circulation Research*.

[33] Pan H., Reilly M. P. (2019). A protective smooth muscle cell transition in atherosclerosis. *Nature Medicine*.

[34] Zhou Z., Chen Y., Zhang D. (2019). MicroRNA-30-3p suppresses inflammatory factor-induced endothelial cell injury by targeting TCF21. *Mediators of Inflammation*.

[35] Blaschuk O. W., Rowlands T. M. (2000). Cadherins as modulators of angiogenesis and the structural integrity of blood vessels. *Cancer Metastasis Reviews*.

[36] Li F., Wan B., Li X. Q. (2022). Expression profile and prognostic values of CDH family members in lung adenocarcinoma. *Disease Markers*.

[37] Saito M., Tucker D. K., Kohlhorst D., Niessen C. M., Kowalczyk A. P. (2012). Classical and desmosomal cadherins at a glance. *Journal of Cell Science*.

[38] Yulis M., Kusters D., Nusrat A. (2018). Cadherins: cellular adhesive molecules serving as signalling mediators. *The Journal of Physiology*.

[39] Niu J., Azfer A., Zhelyabovska O., Fatma S., Kolattukudy P. E. (2008). Monocyte chemotactic protein (MCP)-1 promotes angiogenesis via a novel transcription factor, MCP-1-induced protein (MCPIP). *The Journal of Biological Chemistry*.

[40] Zorniak M., Clark P. A., Kuo J. S. (2015). Myelin-forming cell-specific cadherin-19 is a marker for minimally infiltrative glioblastoma stem-like cells. *Journal of Neurosurgery*.

[41] Zhang Z., Li J., He T., Ding J. (2020). Bioinformatics identified 17 immune genes as prognostic biomarkers for breast cancer: application study based on artificial intelligence algorithms. *Frontiers in Oncology*.

[42] Tippett P., Ellis N. A. (1998). The Xg blood group system: a review. *Transfusion Medicine Reviews*.

[43] Meynet O., Scotlandi K., Pradelli E. (2010). Xg expression in Ewing's sarcoma is of prognostic value and contributes to tumor invasiveness. *Cancer Research*.

[44] Cimino I., Rimmington D., Tung Y. (2021). Murine neuronatin deficiency is associated with a hypervariable food intake and bimodal obesity. *Scientific Reports*.

[45] Yang W., Yuan W., Peng X. (2019). PPAR _*γ*_ /Nnat/NF- _*κ*_ B axis involved in promoting effects of adiponectin on preadipocyte differentiation. *Mediators of Inflammation*.

[46] Li X., Thomason P. A., Withers D. J., Scott J. (2010). Bio-informatics analysis of a gene co-expression module in adipose tissue containing the diet-responsive gene Nnat. *BMC Systems Biology*.

[47] Duan F., Chen X., Yuan L. (2015). Conservation of imprinting of neuronatin (Nnat) in rabbits. *Springerplus*.

[48] Renner M., Wolf T., Meyer H. (2013). Integrative DNA methylation and gene expression analysis in high-grade soft tissue sarcomas. *Genome Biology*.

[49] Uchihara T., Okubo C., Tanaka R. (2007). Neuronatin expression and its clinicopathological significance in pulmonary non-small cell carcinoma. *Journal of Thoracic Oncology*.

[50] Nass N., Walter S., Jechorek D. (2017). High neuronatin (NNAT) expression is associated with poor outcome in breast cancer. *Virchows Archiv*.

[51] Song J., Deng T. (2020). The adipocyte and adaptive immunity. *Frontiers in Immunology*.

