# Supplementary figures and images for: In Silico Identification of Key Genes and Immune Infiltration Characteristics in Epicardial Adipose Tissue from Patients with Coronary Artery Disease
Source: Biomed Res Int. 2022 Oct 29;2022:5610317. doi: 10.1155/2022/5610317 (PMC9637040; doi:10.1155/2022/5610317)

# Supplemental File - Figure 1

A

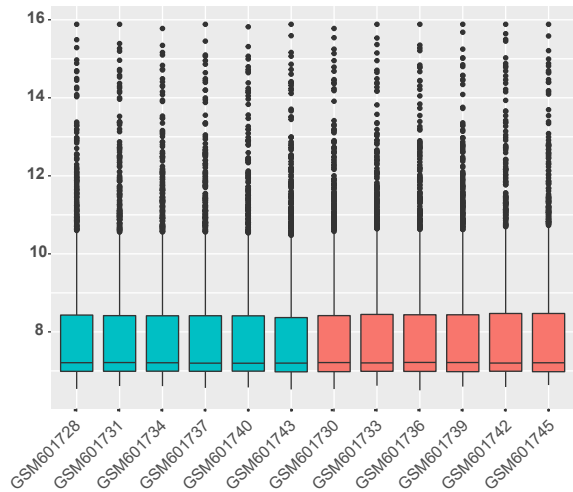

B

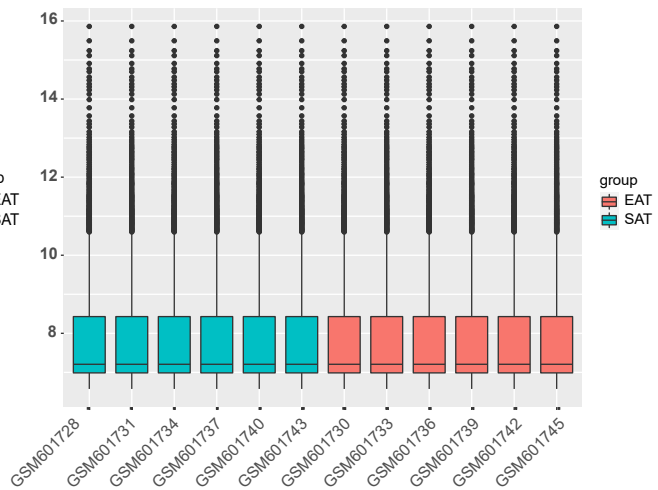

# Supplemental File - Figure 2

A

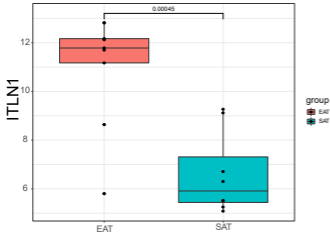

B

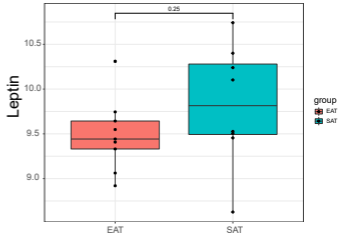

Supplement: Supplementary Materials — Supplemental File Figure 1: box plot of datasets before and after normalization. GSE24425 expression profile before (A) and after (B) normalization. Supplemental File Figure 2: the expression levels of the ITLN1 and leptin in the GSE120774. (A) ITLN1; (B) leptin. [file 5610317.f1.pdf]
